# Comparative Analysis of Dietary Behavior in Children and Parents During COVID-19 Lockdowns in Greece: Insights from a Non-Representative Sample

**DOI:** 10.3390/nu17010112

**Published:** 2024-12-30

**Authors:** Odysseas Androutsos, George Saltaouras, Michail Kipouros, Maria Koutsaki, Athanasios Migdanis, Christos Georgiou, Maria Perperidi, Sousana K. Papadopoulou, Rena I. Kosti, Constantinos Giaginis, Theodora Mouratidou

**Affiliations:** 1 Laboratory of Clinical Nutrition and Dietetics (CND-lab), Department of Nutrition and Dietetics, University of Thessaly, 42132 Trikala, Thessaly, Greece; gsalt@hua.gr (G.S.); mihalis.kip@gmail.com (M.K.); mkoutsaki@uth.gr (M.K.); amigdanis@uth.gr (A.M.); cri.georgiou@gmail.com (C.G.); mperperidi@uth.gr (M.P.); renakosti@uth.gr (R.I.K.); 2 Department of Nutrition and Dietetics, School of Health Sciences and Education, Harokopio University of Athens, 17671 Athens, Greece; 3 Department of Nutritional Sciences and Dietetics, Faculty of Health Sciences, International Hellenic University, 57400 Thessaloniki, Greece; souzpapa@gmail.com; 4 Department of Food Science and Nutrition, School of the Environment, University of the Aegean, 81400 Lemnos, Greece; cgiaginis@aegean.gr; 5 Department of Nutrition and Dietetic Sciences, School of Health Sciences, Hellenic Mediterranean University, 72300 Sitia, Greece; tmouratidou@hmu.gr

**Keywords:** COVID-19, lockdown, nutrition, diet, COVEAT

## Abstract

Background/Objectives: Home isolation measures during the COVID-19 lockdown periods may have influenced individuals’ lifestyles. The COVEAT study aimed to identify differences in children’s and their parents’ dietary behavior, children’s body weight and parental body mass index (BMI) between two lockdown periods implemented in Greece. Methods: In total, 61 participants (children 2–18 years and their parents) completed questionnaires about their lifestyle, body weight and height, and family socio-demographic data, during both lockdown periods (LDs) implemented in Greece (LD1 in March–May 2020; LD2 in December 2020–January 2021). Results: No significant differences in parents’ BMI and fluctuations in children’s/adolescents’ body weight and BMI were observed in LD2 compared to LD1. Regarding dietary behavior, in LD2 fewer parents were found to have dinner and prepared home meals and more families reported to order fast food. Furthermore, a significant decrease in the consumption of fresh and prepacked juices and an increase in fast-food consumption were observed for children/adolescents in LD2 compared to LD1. Conclusions: The findings of the COVEAT study indicate that each lockdown period had a different impact on children’s/adolescents’ and their parents’ dietary behavior, with less favorable changes observed in LD2, suggesting that the implementation of additional lockdowns may have had a negative impact on individuals’ lifestyles.

## 1. Introduction

At the end of 2019, the city of Wuhan, China was confronted with virus of Sars- CoV2 for the first time. The spread of the virus was rapid at a global basis. As a result, on 11 March 2020, the World Health Organization declared that the coronavirus disease 2019 (COVID-19) was a pandemic. In Greece, the first confirmed case of COVID-19 was recorded on 26 February 2020 and in March 2020 the Greek government implemented a nationwide closure of educational institutions of all levels and all public areas, such as cafes, restaurants, shopping centers, and churches. On 23 March 2020, the first national lockdown was implemented and only certain commuting activities (e.g., walking, cycling) and work were allowed upon individuals’ request and the approval of the national authorities. On 5 November 2020, the government announced the second national lockdown and the relevant regulations for community activities and work were reactivated. The lockdown period in Greece ended in early 2021 with the reopening of the preschools and primary schools on 11 January 2021 and secondary schools on February 1st and the termination of the restrictions for all age groups.

The implementation of lockdowns led to changes in individuals’ lifestyles, previously described [1,2,3]. Specifically, during the first lockdown, where the fear of this unknown disease prevailed, citizens tended to have higher compliance with the home isolation measures [4]. Previous studies have shown that this home isolation led to significant changes in eating behavior [3,4]. Two studies reported that children, adolescents, and young adults increased their consumption of healthy foods such as fruits, vegetables, and juices with simultaneous reduction in fast-food consumption. In addition, an increase in sedentary time and a decrease in physical activity level were recorded, even though citizens had the opportunity to perform outdoor physical activities [5,6,7]. Similar findings were observed in the second lockdown. Some studies reported that during the second lockdown, individuals increased healthy foods, mostly fresh fruits and vegetables, and decreased processed foods and added sugar, compared to the first lockdown [8]. However, other studies revealed contradictory findings, indicating that certain lifestyle indices, including diet quality, worsened during the second lockdown [9].

Therefore, this study aims to explore the variations in dietary behavior and body mass index (BMI) between the first and second lockdowns in children, adolescents, and their parents in Greece, addressing a critical gap in the existing literature.

## 2. Materials and Methods

### 2.1. Study Design and Participants

The COVEAT study had a cross-sectional design. Its detailed protocol has been previously presented elsewhere [10]. In brief, families (parents and their children aged 2–18 years) from 63 municipalities in Greece were recruited from networks of dietitians-nutritionists, personal networks, and social media and provided data in two time-points: in March–May 2020 (1st lockdown) and in December 2020–January 2021 (2nd lockdown).

This study adhered to the Declaration of Helsinki and the conventions of the Council of Europe on Human Rights and Biomedicine; received ethical clearance from the Ethical Committee of the Department of Physical Education and Sport Science, University of Thessaly; and was registered at clinicaltrials.org (NCT04437121). Prior to their enrollment in this study, participants signed an online informed consent form.

### 2.2. Questionnaires

The questionnaires, based on a Food Frequency Questionnaire (FFQ), were completed online at both lockdowns, using Google-forms. Τhese questionnaires collected data on family sociodemographic characteristics (e.g., age, family composition, region of residence, parental occupation, and educational level), anthropometric indices (e.g., parental and children’s/adolescents’ weight and height) and parents’ and children’s/adolescents’ lifestyle behaviors (e.g., main meals, snacks and fast-food consumption, and consumption of various foods). More specifically, the frequency of each main meal (breakfast, lunch, dinner) was self-reported by the participants and variables were then dichotomized to “Yes” (for those consuming each meal every day) and “No” (for the rest of participants). Furthermore, the frequencies and servings of each food (fruit, fresh juices, prepacked juices, vegetables, dairy, red meat, poultry, fish, pasta, legumes, homemade sweets, ready-made sweets, salty snacks, fast food, and soda beverages) was also self-reported by the participants and servings per week of each food were calculated. All dietary intake data were collected retrospectively for the lockdown periods, with no prospective meal recordings. Parents (one partner: husband or wife) provided data on behalf of their children, reporting both dietary intake and anthropometric measurements. Each family contributed data for one child only. Body mass index (BMI) was calculated according to the following equation: Weight (kg)/ Height (m^2^).

### 2.3. Statistical Analysis

Continuous data are presented as mean ± standard deviation (SD). Categorical variables are presented as absolute (n) and relative (%) frequencies. The variables “preparing meal at home” and “ordering fast food” were transformed from ordinal to continuous. For paired comparisons of continuous data that were normally distributed (Shapiro–Wilk test for evaluation of data distribution), Student’s test for paired data was applied. For paired comparisons of continuous data that were not normally distributed and for categorical data, the Wilcoxon matched-pairs signed-rank test was applied. The level of statistical significance was set to *p* < 0.05 for analyses. All analyses were performed with SPSS V26 software package (IBM, Armonk, NY, USA).

## 3. Results

In total, 61 participants completed the survey during both the 1st and the 2nd lockdown. Table 1 summarizes the demographic and occupational characteristics of the study participants, providing critical context for interpreting the findings. Most surveys were completed by females/mothers (93.4% for both lockdowns). The table reveals educational differences among parents: 39.3% of fathers and 19.7% of mothers had completed secondary school, while 21.3% of fathers and 31.1% of mothers held postgraduate qualifications. Family status data indicate that 95.1% of participants came from families with married parents, and 4.9% were from single-parent households. Employment changes during the lockdowns varied significantly between fathers and mothers. Among fathers, 37.7% reported no change in employment status, while 29.5% experienced reduced working hours. In contrast, 49.2% of mothers experienced no employment changes, while 16.4% reported reduced hours. Additionally, fathers were more likely to work from home (8.2% compared to 4.9% for mothers) and to leave for personal or exceptional reasons (13.1% versus 8.2% for mothers).

**Figure 1:**
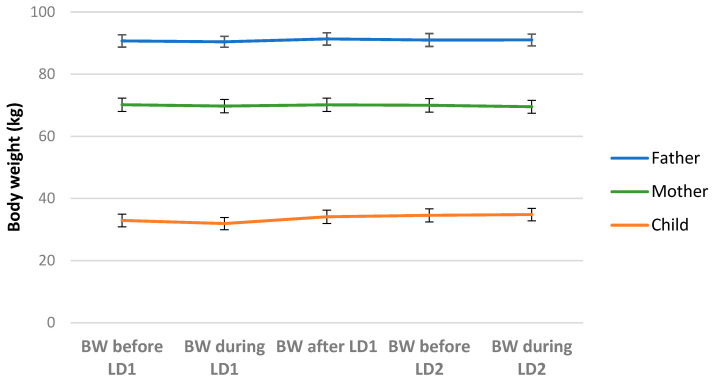
Changes in parents’ and children’s body weight at different time points (before first COVID-19 lockdown until during second COVID-19 lockdown). BW: Body weight; LD1: First COVID-19 lockdown; LD2: Second COVID-19 lockdown. Data presented as mean (SE). *p* < 0.05 for all comparisons (child/adolescent): BW before LD1 vs. BW during LD1, BW during LD1 vs. BW after LD1, BW after LD1 vs. BW before LD2, BW before LD2 vs. BW during LD2, BW during LD1 vs. BW before LD2.

At baseline, mean (SD) age was 43.0 (6.6) years and 39.9 (5.7) years for fathers and mothers, respectively, and 7.7 (4.2) for their children. Table 2 presents the anthropometric characteristics of parents and children/adolescents during both lockdowns, and Table 1 presents the baseline demographic characteristics of the parents during the 1st lockdown. Both parents’ weight (Figure 1) and body mass index (ΒΜΙ) were similar during lockdowns and did not fluctuate during the period before the imposing of the 1st lockdown until the second lockdown (*p* > 0.05 for all comparisons). On the other hand, there was a significant increase in children’s/adolescents’ body weight and body mass index (Table 2; Figure 1). Interestingly, there was a significant decrease in children’s/adolescents’ body weight during the first lockdown, compared to the period before the first COVID-19 lockdown (mean difference 1.14 kg; *p* < 0.001).

In relation to changes in dietary habits and practices, there were no changes in the consumption of all three main meals (breakfast, lunch, and dinner) with the exception of a decreased percentage of parents reporting having dinner during the second COVID-19 lockdown (*p* = 0.025). Similarly, no changes were detected in the consumption of snacks for either parents or children (Table 3). There was a significant increase in ordering/consuming fast foods for both parents and children (*p* < 0.001). Parents ordered/consumed fast foods 0.64 times/week during LD1 and 1.17 times/week during LD2 (*p* < 0.001), while children consumed fast foods 0.55 and 1.08 times/week, respectively (*p* < 0.001). Parents also reported preparing home meals less often during LD2, compared to LD1 (6.09 times/week vs. 6.43 times/week; *p* = 0.027).

Table 4 presents the changes in the consumption of different food groups (available only for children). There was a significant decrease in the weekly consumption of fresh and prepacked juices (−1 and −0.47 servings/week, respectively) and fish (−0.14 servings/week) and a significant increase in weekly consumption of fast foods (+0.53 servings/week).

## 4. Discussion

The COVEAT study assessed differences in parents’ and their children’s BMI or body weight, respectively, and their dietary behavior between the two lockdown periods implemented in Greece. The results of this study did not identify any significant changes in fathers’ and mothers’ parental BMI. However, significant changes in children’s /adolescents’ body weight were recorded during the second lockdown (LD2) compared to the first lockdown (LD1). Regarding dietary behavior, fewer parents were found to prepare home meals in LD2, and they increased fast-food consumption. In addition, a significant decrease in fresh and prepacked juices consumption and an increase in fast-food consumption were recorded in children/adolescents in LD2 compared to LD1.

Only a few studies focused on individuals’ change in dietary behavior and anthropometric indices between different lockdown periods. The vast majority of the studies are based on data obtained in LD1. The study of Parker et al. observed that over 40% of adults 18–28 years old living in the United States of America had an increase and approximately 17% a decrease in their body weight. Interestingly, a higher increase occurred among the lower income participants and in females [11]. The study by Al Zaman et al. included 439 adults 18–59 years old in the United Arab Emirates. Based on the results of this study, 51.1% of the participants gained weight, 36.2% lost weight, and 12.7% maintained their body weight [12]. Rizzo et al. presented the results of a large-scale, online study in 140 countries (n = 19.903) and showed that a large number of citizens, especially in the USA, increased their body weight during LD1 [13]. In addition, Khan et al. conducted a review of studies (n = 41) and concluded that body weight increased in LD1 in 6–8-year-old children, especially in those who were already living with overweight or obesity. On the other hand, Bahatheg conducted a study with 330 parents from three countries (Great Britain, Saudi Arabia, and Turkey) and revealed that 63% of the parents reported that their children did not gain weight in LD1 [14].

Regarding the changes in dietary behavior in LD1, the studies by Phillippe et al. and Caso et al. reported a significant increase in healthy food consumption in parents and their children and preparation of home meals [15,16]. Moreover, Phillippe et al. observed that parents preferred to prepare local recipes and to use raw materials and Caso et al. observed a decrease in adults’ fast-food consumption [15,16]. During LD1, Gaa et al. recorded a significant increase in homemade foods consumption and a decrease in meal skipping in a sample of 220 students in Uganda [17]. Regarding the number of main meals, Bahatheg showed that the majority of participants consumed three main meals per day (59.7%) and prepared those meals at home, but also captured an increase in consumption of soft drinks, sweetened juices, juice blends, fruit juice, and frozen food (e.g., pizza, nuggets) in children [14]. Furthermore, the study revealed that a high number of children were eating meat and drinking milk, but fewer were consuming fish. Gomes et al. reported minor changes in fruits, vegetables, and ultra-processed foods consumption in Brazilian adolescents [18]. On the other hand, the study of Parker et al. reported an increase in fast-food consumption, especially among young adults with lower incomes, while the high-income participants had a significant increase in homemade food consumption [11].

Previous studies have also explored individuals’ changes of lifestyle and BMI in LD2. According to Bell et al., there was a significant increase in self-reported body mass and BMI at LD1 compared to LD2 [9]. Additionally, there was an increase in the proportion of participants meeting the criteria for overweight between LD1 and LD2 [9]. Comparable results were reported in the study by Kriaučionienė et al., where the most significant increase in BMI was observed in participants who were already overweight. According to the changes in dietary behaviors, this study observed a significant increase in fast-food consumption among both parents and children in LD2 compared to LD1. Furthermore, a decline in the preparation of homemade meals was recorded, although no significant change was observed. A reduction in dinner consumption was accompanied by a simultaneous decrease in the intake of both fresh and prepackaged juices, as well as fish. In accordance with the findings of Kriaučionienė et al., a significant increase in the consumption of fast food, sugary beverages, along with a greater volume of food ordered for home delivery or takeaway, was strongly correlated with weight gain in more than 60% of the participants [19]. According to Pfiefer et al., during LD2 the consumption of certain unhealthy foods (e.g., processed meat products and sweets) was observed among Croatian students [20]. In accordance with the aforementioned, the study by Abdelkawy also reported that participants under 20 years old increased their consumption of carbonated beverages, commercial pastries, fried foods, and fast food, along with difficulties in finding certain foods such as honey, olive oil, broccoli, oranges, and pineapples [21].

The differences observed in individuals’ dietary behaviors between LD1 and LD2 could be attributed to the limited knowledge regarding the health effects of COVID-19 during LD1, which in combination with the home isolation measures seem to have led them in adopting a healthier dietary pattern. More specifically, Di Renzo et al. [4] highlighted that the initial fear of infection during LD1 motivated individuals to focus on healthier behaviors, such as preparing meals at home and consuming fresh foods. However, the progressive normalization of the pandemic and the opening of retail and food services during LD2 facilitated the higher consumption of fast food [19,22]. Understanding the differences in dietary behavior between LD1 and LD2 provides crucial insights for public health strategies. The findings suggest that increased stress and lifestyle changes during the second lockdown were associated with less healthy dietary choices, such as a higher consumption of fast- food. These results can inform targeted interventions aimed at promoting home meal preparation and healthy food consumption, particularly during health crises. By providing educational tools and support to families, future strategies could mitigate the impact of restrictions on diet and weight, fostering long-term health and well-being.

The findings of the present study should be interpreted under the light of its strengths and limitations. The COVEAT study was the first study in Greece which investigated the differences in dietary behavior in parents and children/adolescents and the changes in their body weight and BMI during LD1 and LD2. As a result, its methodological design offers the opportunity to obtain some insights in this field, using data obtained by the same study participants in both lockdown periods. The limitations of this study focus on the small, non-representative sample size and the fact that participants were mainly females and well-educated participants; therefore, the results may not be generalizable to the general population. Furthermore, given the restrictions which were in place during the lockdown periods, the data were collected via networks of dietitians-nutritionists which might have caused selection bias. Also, data were based on self-reported questionnaires, since objective methods (e.g., anthropometric measurements such as body weight and height) could not be applied. Finally, due to the limited number of participants, it was not possible to conduct additional statistical analyses to identify differences among sub-groups of the population which may have been affected more, due to the home isolation, such as individuals with different socio-economic backgrounds, weight categories, genders, age groups, health status, etc. Future meta-analyses should aim to explore differences in citizens’ lifestyle behaviors and health indices among the lockdown periods, using larger sample sizes from pre-existing cohorts to better understand the impact that home isolation had in individuals’ health status.

## 5. Conclusions

The COVEAT study revealed that dietary behaviors varied across the different lockdown periods implemented in Greece. The findings suggest that each lockdown period had a different impact on individuals’ (children’s/adolescent’ and parents’) dietary behaviors. Notably, more unfavorable changes were observed during LD2, indicating that the potential implementation of additional lockdowns could negatively affect individuals’ lifestyle choices. The changes in 61 individuals’ dietary patterns during the lockdowns implemented during the COVID-era underscore the need of applying careful monitoring and lifestyle interventions to prevent long-term negative impacts on individuals’ health status and well-being, particularly among vulnerable population groups. Further research with larger and more diverse populations is necessary to validate these results and ensure that they are representative of broader groups.

## Figures and Tables

**Table 1:** Parental demographic characteristics (n = 61).

Characteristics		N (%)
Father’s education	Primary school	2 (3.3)
	Secondary school	24 (39.3)
	Clerical/Commercial/Professional qualification (IEK)	5 (8.2)
	University qualification	17 (27.9)
	Postgraduate qualification	13 (21.3)
Mother’s education	Primary school	1 (1.6)
	Secondary school	12 (19.7)
	Clerical/Commercial/Professional qualification (IEK)	4 (6.6)
	University qualification	25 (41)
	Postgraduate qualification	19 (31.1)
Family status	Married parents	58 (95.1)
	Single-parent families	3 (4.9)
Changes in father’s job during lockdown	No change	23 (37.7)
	Work from home	5 (8.2)
	Increased hours at work	1 (1.6)
	Reduced hours at work	18 (29.5)
	Leave for personal or exceptional reasons	8 (13.1)
	Unemployed	3 (4.9)
	Other	3 (4.9)
Changes in mother’s job during lockdown	No change	30 (49.2)
	Work from home	3 (4.9)
	Increased hours at work	1 (1.6)
	Reduced hours at work	10 (16.4)
	Leave for personal or exceptional reasons	5 (8.2)
	Unemployed	2 (3.3)
	Other	10 (16.4)

**Table 2:** Anthropometric characteristics for parents and child/adolescent.

Characteristics	LD1 (Mean (SD))	LD2 (Mean (SD))	*p* Value
Father’s weight (kg) (n = 57)	90.4 (13.5)	91.0 (14.4)	0.294
Father’s body mass index (kg/m^2^) (n = 57)	28.4 (4.3)	28.7 (4.2)	0.239
Mother’s weight (kg) (n = 61)	69.7 (16.7)	69.5 (16.3)	0.880
Mother’s body mass index (kg/m^2^) (n = 57)	25.6 (5.1)	25.6 (4.8)	0.179
Child/Adolescent’s weight (n = 60)	31.9 (15.4)	34.8 (15.7)	<0.001

SD: Standard Deviation; LD1: First COVID-19 lockdown; LD2: Second COVID-19 lockdown.

**Table 3:** Dietary practices for parents and children during the first and second COVID-19 lockdown (n = 61).

Practices		Parent			Child		
		LD1	LD2	*p* Value	LD1	LD2	*p* Value
		N (%)	N (%)		N (%)	N (%)	
Consumption of breakfast	Yes	58 (95.1)	54 (88.5)	0.157	60 (98.4)	58 (95.1)	0.317
Consumption of lunch	Yes	59 (96.7)	61 (100)	0.157	61 (100)	60 (98.4)	0.317
Consumption of dinner	Yes	60 (98.4)	55 (90.2)	0.025	59 (96.7)	57 (93.4)	0.414
Consumption of snacks	No snack	0 (0)	0 (0)	0.826	1 (1.6)	1 (1.6)	0.854
	1	12 (19.7)	12 (19.7)		7 (11.5)	7 (11.5)	
	2	30 (49.2)	30 (49.2)		31 (50.8)	31 (50.8)	
	3	13 (21.3)	15 (24.6)		14 (23.0)	15 (24.6)	
	>=4	6 (9.8)	4 (6.6)		8 (13.1)	7 (11.5)	
Ordering/eating fast food	Never	27 (44.3)	10 (16.4)	<0.001 *	30 (49.2)	10 (16.4)	<0.001 *
	1–3 times/month	18 (29.5)	21 (34.4)		19 (31.1)	25 (41.0)	
	1–2 times/week	13 (21.3)	22 (36.1)		9 (14.8)	20 (32.8)	
	3–4 times/week	3 (4.9)	8 (13.1)		3 (4.9)	5 (8.2)	
	5–6 times/week	0 (0)	0 (0)		0 (0)	1 (1.6)	
	Every day	0 (0)	0 (0)		0 (0)	0 (0)	

LD1: First COVID-19 lockdown; LD2: Second COVID-19 lockdown; * Paired *t*-tests for continuous variables, as mentioned in text (transformed from nominal variables).

**Table 4:** Children’s and adolescents’ eating habits * during the first and second COVID-19 lockdown (n = 60).

Food Groups	LD1	LD2	*p* Value
Fruits	5.11 (2.32)	4.92 (2.30)	0.591
Fresh juices	4.03 (2.48)	3.03 (2.38)	0.004
Prepacked juices	1.50 (2.01)	1.03 (1.72)	0.044
Vegetables	4.15 (2.65)	4.00 (2.56)	0.836
Dairy	6.52 (1.45)	6.34 (1.38)	0.401
Red meat	2.12 (1.27)	2.16 (1.15)	0.704
Poultry	1.65 (0.73)	1.66 (0.98)	0.902
Fish	1.21 (0.52)	1.07 (0.73)	0.035
Pasta	3.60 (2.09)	3.74 (1.97)	0.490
Legumes	1.46 (0.94)	1.43 (0.73)	0.783
Homemade sweets	2.55 (2.06)	2.39 (2.14)	0.533
Ready-made sweets	2.51 (2.31)	2.65 (2.29)	0.669
Salty snacks	1.21 (1.61)	1.29 (1.59)	0.679
Fast food	0.55 (0.85)	1.08 (1.09)	<0.001
Soda beverages	0.71 (1.63)	0.58 (1.39)	0.205

* Data presented as mean (SD) consumption (in servings/week) of each food. LD1: First lockdown; LD2: Second lockdown.

## Data Availability

The data presented in this study are available on request from the corresponding author. The data are not publicly available due to ethical restrictions.

## References

[1] Naja F., Hamadeh R. (2020). Nutrition amid the COVID-19 pandemic: A multi-level framework for action. Eur. J. Clin. Nutr..

[2] Butler M.J., Barrientos R.M. (2020). The impact of nutrition on COVID-19 susceptibility and long-term consequences. Brain Behav. Immun..

[3] Pietrobelli A., Pecoraro L., Ferruzzi A., Heo M., Faith M., Zoller T., Antoniazzi F., Piacentini G., Fearnbach S.N., Heymsfield S.B. (2020). Effects of COVID-19 Lockdown on Lifestyle Behaviors in Children with Obesity Living in Verona, Italy: A Longitudinal Study. Obesity.

[4] Di Renzo L., Gualtieri P., Pivari F., Soldati L., Attinà A., Cinelli G., Leggeri C., Caparello G., Barrea L., Scerbo F. (2020). Eating habits and lifestyle changes during COVID-19 lockdown: An Italian survey. J. Transl. Med..

[5] Robinson T.N., Banda J.A., Hale L., Robinson T.N., Banda J.A., Hale L., Lu A.S., Fleming-Milici F., Calvert S.L., Wartella E. (2017). Screen Media Exposure and Obesity in Children and Adolescents. Pediatrics.

[6] Tambalis K.D., Panagiotakos D.B., Psarra G., Sidossis L.S. (2018). Insufficient Sleep Duration Is Associated with Dietary Habits, Screen Time and Obesity in Children. J. Clin. Sleep Med..

[7] Avery A., Anderson C., McCullough F. (2017). Associations between children’s diet quality and watching television during meal or snack consumption: A systematic review. Matern. Child Nutr..

[8] Woods N., Seabrook J.A., Schaafsma H., Burke S., Tucker T., Gilliland J. (2024). Dietary changes of youth during the COVID-19 pandemic: A systematic review. J. Nutr..

[9] Bell M., Duncan M.J., Patte K.A., Roy B.D., Ditor D.S., Klentrou P. (2023). Changes in Body Mass, Physical Activity, and Dietary Intake during the COVID-19 Pandemic Lockdowns in Canadian University Students. Biology.

[10] Androutsos O., Perperidi M., Georgiou C., Chouliaras G. (2021). Lifestyle Changes and Determinants of Children’s and Adolescents’ Body Weight Increase during the First COVID-19 Lockdown in Greece: The COV-EAT Study. Nutrients.

[11] Parker J., Kaur S., Medalla J.M., Imbert-Sanchez A., Bautista J. (2023). Dietary trends among young adults during the COVID-19 lockdown: Socioeconomic and gender disparities. BMC Nutr..

[12] Zaman K.A., Ahmed S., Alshamsi A., Alshamsi A., Alshdaifat B., Alaleeli S., Mussa B.M. (2023). Impact of COVID-19 Pandemic on Weight Change Among Adults in the UAE. Int. J. Gen. Med..

[13] Rizzo N. (2021). Quarantine Weight Gain: 35.82% Gained Weight During Pandemic [19,903 Person Study]. https://runrepeat.com/quarantine-15-weight-gain-study.

[14] Bahatheg R.O. (2021). Young Children’s Nutrition During the COVID-19 Pandemic Lockdown: A Comparative Study. Early Child. Educ. J..

[15] Philippe K., Issanchou S., Monnery-Patris S. (2021). Contrasts and ambivalences in French parents’ experiences regarding changes in eating and cooking behaviours during the COVID-19 lockdown. Food Qual. Prefer..

[16] Caso D., Guidetti M., Capasso M., Cavazza N. (2021). Finally, the chance to eat healthily: Longitudinal study about food consumption during and after the first COVID-19 lockdown in Italy. Food Qual. Prefer..

[17] Gaa P.K., Sulley S., Boahen S., Bogobiri S., Mogre V. (2022). Reported dietary habits and lifestyle behaviors of students before and during COVID-19 lockdown: A cross-sectional survey among university students from Ghana. J. Public Health Res..

[18] Gomes C.S., Santi N.M.M., da Silva D.R.P., Werneck A.O., Szwarcwald C.L., de Azevedo Barros M.B., Malta D.C. (2022). The COVID-19 pandemic and changes in eating habits of Brazilian adolescents. Dialogues Health.

[19] Kriaučionienė V., Grincaitė M., Raskilienė A., Petkevičienė J. (2023). Changes in Nutrition, Physical Activity, and Body Weight among Lithuanian Students during and after the COVID-19 Pandemic. Nutrients.

[20] Pfeifer D., Rešetar J., Czlapka-Matyasik M., Bykowska-Derda A., Kolay E., Stelcer B., Gajdoš Kljusurić J. (2023). Changes in diet quality and its association with students’ mental state during two COVID-19 lockdowns in Croatia. Nutr. Health.

[21] Abdelkawy K., Elbarbry F., El-Masry S.M., Zakaria A.Y., Rodríguez-Pérez C., El-Khodary N.M. (2023). Changes in dietary habits during Covid-19 lockdown in Egypt: The Egyptian COVIDiet study. BMC Public Health.

[22] Bohlouli J., Moravejolahkami A.R., Ganjali Dashti M., Balouch Zehi Z., Hojjati Kermani M.A., Borzoo-Isfahani M., Bahreini-Esfahani N. (2021). COVID-19 and fast foods consumption: A review. Int. J. Food Prop..

